# Neuromodulation for Pain Management in the Inpatient Setting: A Narrative Review

**DOI:** 10.7759/cureus.13892

**Published:** 2021-03-15

**Authors:** Alaa Abd-Elsayed, Tuan Tang, Jay Karri, Meghan Hughes, Ivan Urits, Mayank Gupta, Alberto Pasqualucci, Dariusz Myrcik, Giustino Varrassi, Omar Viswanath

**Affiliations:** 1 Anesthesiology and Pain Management, University of Wisconsin, Madison, USA; 2 Anesthesiology and Critical Care, University of Texas at Houston, Houston, USA; 3 Anesthesiology, Baylor College of Medicine, Houston, USA; 4 Anesthesia, University of Wisconsin School of Medicine and Public Health, Madison, USA; 5 Department of Anesthesia, Critical Care and Pain Medicine, Beth Israel Deaconess Medical Center, Harvard Medical School, Boston, USA; 6 Pain Management, Kansas City University of Medicine and Biosciences, Kansas City, USA; 7 Anesthesia and Critical Care, University of Perugia, Perugia, ITA; 8 Emergency, Medical University of Silesia, Bytom, POL; 9 Research, Paolo Procacci Foundation, Rome, ITA; 10 Pain Management, Creighton University School of Medicine, Phoenix, USA

**Keywords:** neuromodulation, inpatient and outpatient setting, tens, pns, tdcs, rtms, pemf

## Abstract

Pain is highly prevalent and pharmacological therapy is not always efficacious. There are a few pathophysiological reasons to believe that neuromodulation would increase the rate of success of pain management. This review article is focused on that aspect, discussing non-invasive or minimally invasive neuromodulation techniques in both the inpatient and outpatient setting. This article provides an in-depth discussion of the multiple neuromodulation techniques available over time to be suitable and effective when used as analgesic therapies for chronic pain. We reviewed the literature and discussed all available neuromodulation options that were tested in the inpatient and outpatient setting. Neuromodulation plays a very important role in treating chronic pain in both inpatient and outpatient setting.

## Introduction and background

Chronic pain is a complex and costly condition that often does not have a clear clinical picture [1]. Currently, only 40-60% of patients suffering from chronic pain syndromes report favorable outcomes from the pharmacologic therapies [2,3], with side effects of analgesic medications being one of the reasons [4-6]. Moreover, studies consistently indicate that the majority of available treatments for chronic pain such as antidepressants, opioids and topical anesthetics exhibit limited long-term effectiveness and can often be “associated with moderate or in some cases, severe adverse effects” [7]. Furthermore, the current approaches (often pharmacologic) to treating chronic pain have a limited to no effect on the mechanisms underlying chronic pain [8-10]. Patients may suffer for several years before they successfully find a doctor who can potentially address their suffering.

The majority of existing pain research has been dedicated to understanding the genetic components of this condition. However, advances in imaging and new research have elucidated the fact that pain also has a “neural circuit-based signature” which can be exploited for therapeutic purposes. As a result, “neuromodulation has emerged as a specific form of treatment without the systemic side effects of pharmacotherapies” [11]. Neuromodulation has many strengths: “reversibility, programmability, low risk and specificity” [12]. The benefits of neuromodulation include improved quality of life, relief from pain, and enhanced functional status [13]. All of these correspond to a reduction in overall healthcare needs and utilization of healthcare resources.

Although neuromodulation has consistently demonstrated efficacy as a therapy for pain refractory to traditional medical management or surgery and has become a therapy of choice for many practitioners, the scientific evidence around the technique remains modest. For this reason, further high-level scientific studies must be conducted to officially justify its application among patients, practitioners, and payers alike. In addition, there is a need to generate awareness and uptake among patients, caretakers, and physicians involved [12].

In this paper, we will provide detailed analysis and discussion on some of the most common neuromodulation techniques currently utilized to treat chronic, refractory pain: transcranial direct current stimulation (tDCS), transcutaneous electrical nerve stimulation (TENS), repetitive transcranial magnetic stimulation (rTMS), pulsed electromagnetic field (PEMF), and peripheral nerve stimulation (PNS).

## Review

Transcranial direct current stimulation

Overview

tDCS is a non-invasive cortico-neuromodulatory stimulation treatment that utilizes a constant low-intensity electrical current to modulate neuronal activity [13-15]. By applying anodal or cathodal currents via electrodes positioned on the scalp, clinicians can target regional areas of the brain and produce subthreshold excitatory or inhibitory effects on the resting membrane potential based on the polarity of the electrode and can enhance or weaken synaptic connections [16].

tDCS “has a theoretical advantage when compared with traditional chronic pain treatments since it directly affects central neural targets” [17]; thus, possibly more effectively addressing central chronic pain which is believed to be a byproduct of maladaptive plasticity within the pain pathway. tDCS combined with other behavioral interventions can be used to augment synaptic plasticity and long-term potentiation [18]. tDCS has been reported to decrease pain sensations while increasing pain threshold, with some studies citing a mean percentage of improvement to be greater than 60% [18]. It should be recognized that tDCS can take longer to manifest its effects, typically after multiple sessions [19].

Candidacy

The efficacy of tDCS has been studied at length in recent years with multiple meta-analyses being performed on the multitude of studies reported. It must be made clear that all of these studies are underpowered, albeit with promising results. There is a high variability in the application, protocol, and utilization of tDCS. Positive results have emerged from many of these studies; however, “clinical recommendations have been made for only two pain conditions: fibromyalgia (level B of evidence) and lower limb pain due to spinal cord injury (level C evidence)” [20].

Considering that tDCS is non-invasive, portable, inexpensive, and generally harmless, there are few barriers to actual application outside of an official medical indication. Perhaps the greatest challenge is the selection of patients to treat. It currently remains difficult to predict which patients will respond to the treatment. Therefore, there is a growing practice of designating patients as “responders” or “non-responders” and then backing out of these designations to explore possible response predictors retrospectively with the hope of eventually parlaying this into prospective predictions.

Conditioned pain modulation (CPM) is believed to be a biomarker for chronic pain which could be used across all types of neuromodulatory modalities. Unfortunately, the problems are quite significant. That is, there are no normative data, and a “normal range” has yet to be established, and it is clear that there is significant interindividual variance with CPM magnitude that is likely tied to qualifiers such as age, sex, and other undiscovered variables. “As an example, fibromyalgia (FM) patients demonstrated a lower conditioned pain modulation (CPM) activity since the rate of FM patients that report pain facilitation during CPM assessments is significantly increased compared with controls (41.7% vs 21.2%)” [21]. Taking this data into consideration while extending on the theory that a high CPM response (high pathway impairment) is indicative of high response to tDCS, one could gestalt that patients with high CPM response will also be patients with a strong response to tDCS. Regardless, what is agreed upon and clearly needed are further studies with stricter adherence to consistent, ubiquitous protocols.

Exclusion criteria for candidacy include (but are not limited to) pregnancy, difficulty with scalp contact including hair quality or skin conditions, metallic implants, recent head trauma, prior seizure history, or a history of adverse side effects with prior brain stimulation.

Anatomy

During central pain processing, constellations or matrixes of various neural sites are activated, which can be collectively known as pain neuromatrices [18]. The superficial pain neutomatrix includes the primary sensory cortex (S1), primary motor cortex (M1) and dorsolateral prefrontal cortex; while the deeper pain neuromatrix include the thalamus, insula, anterior cingulate cortex, and pre-aqueductal grey matter. Targets of interest for tDCS have included S1 and M1 of the superficial PNM. Within the pain pathway, the S1 is responsible for nociceptive sensory discrimination such as pain localization, intensity, and quality or characteristic [15].

Procedure

By placing two electrodes of opposite polarity, a circuit is rendered across the brain [18]. To stimulate the motor cortex unihemispherically, the positive anode electrode can be placed contralateral to the painful somatic area, while the negative cathode can be placed on the contralateral supraorbital region. Wet saline sponge or electro-conductive gel is required for optimal cutaneous contact and thus conductivity. While it is not easy to determine effective design due to a lack of large evidence-based randomized control trials and a large variation in tDCS protocols between studies, commonly a current between 1 and 2 mA is applied ranging from 5-30 minutes with electrode size of 25-35 cm^2^ [15]. Localization or position of electrode placement can be based on physiologic/anatomical placement, which may be used to target more superficial cortices such as the M1. More intricate or detailed targets can be obtained via the 10:20 electroencephalogram system.

Potential Complications

Currently, there is no evidence to support significant adverse effects including changes in cognitive performance or effects on brainstem autonomic function during or after stimulation as long as the current is below the safety threshold of 2 mA [14]. Most common complaints include skin irritation at the electrode site as well as cutaneous sensations during or after tDCS activity, which are mild and transient. However, improper placement and administration can cause site-dependent injuries such as skin burn or ocular sensation. Variables that alter current flow include structural (e.g., hair thickness, head size, skull thickness), biological (e.g., gamma aminobutyric acid neurotransmitter levels, age), and lifestyle (e.g., neuroaffective substances intake, personality) factors [15].

Transcutaneous electrical nerve stimulation

Overview

TENS is a non-invasive neuromodulatory technique that utilizes electrical currents delivered on the skin through electrodes to dermatomal sites for potential localized analgesia [22,23]. The main modalities of TENS include conventional TENS, which uses high-frequency, low-intensity electrical current to inhibit the segmental pathway, and acupuncture-like TENS (ALTENS), which uses low-frequency, high-intensity electrical current to activate the inhibitory extra-segmental pathway (Table 1). TENS modifies hyperalgesia through two mechanisms: afferent inputs to the central nervous system as well as restoration of central pain modulation as seen in the treatment of fibromyalgia.

The last decade has seen numerous systematic reviews and meta-analyses examining the efficacy of TENS related to a multitude of conditions such as neck pain [24], cancer pain [25], acute pain [26], and low back pain [27]. The bottom line is that the results of all these studies are conflicting at best. Many times, the challenges faced are related to the quality of the studies themselves as well as a lack of consistency related to the methodology of the TENS therapy between different providers. At the very least, TENS therapy requires further, high-quality research [28]. Of note, there is consensus around factors that primarily the efficacy of TENS: (1) tolerance to repeated TENS; (2) intensity of the stimulation; and (3) electrode placement [29]. Other considered indications explored have yielded weak evidence for labor pain, insufficient evidence for dysmenorrhea, and inconclusive evidence for chronic pain; therefore, further investigation is warranted to verify any benefits of TENS [22].

**Table 1:** Different characteristics and mechanisms of action of TENS. TENS, transcutaneous electrical nerve stimulation; CNS, central nervous system

	Characteristics*	Mechanism of action
Conventional TENS	High-frequency (50–100 Hz), low-intensity, small pulse width (50–200 μs)	Stimulate large diameter, low threshold non-noxious afferent (A-beta) in dermatomes, which inhibits the segmental pathway (via inactivation of nociceptors and sensitization in the CNS)
Acupuncture-Like TENS	Low-frequency (2–4 Hz), high intensity, long pulse width (100–400 μs)	Stimulate small diameter, high threshold peripheral afferent (A-delta) fibers, which activates the inhibitory extra-segmental pathway (via activation of midbrain peri-aqueductal grey and rostral ventromedial medulla)

Candidacy

Similar to other forms of neuromodulation, the indication for TENS therapy would be central pain symptoms. However, it is better suited to address localized central pain [21,30]. Because of its cutaneous approach, TENS should not be used on open wounds, skin pathologies including malignancies, or non-sensate dermatomal areas affected by tactile allodynia and hyperaesthesia [22,26]. Important dangerous considerations for electrode placement location include carotid sinus stimulation, laryngeal spasms, increased ocular pressure, and in the anterior and posterior chest include cardiac conduction abnormalities and pulmonary compromise via intercostal muscles overstimulation. Contraindications for electrotherapies such as TENS include cardiac pacemakers and implantable cardioverter-defibrillators, pregnancy, and epilepsy. However, TENS use can be considered in certain situations under professional supervision [30,31].

Anatomy

Electrodes should be placed on healthy skin without neuropathies on the dermatomes of interest. Alternative placements can include larger proximal nerves that supply distal innervation or paravertebral to the corresponding spinal segment.

Procedure

After ensure that the contact site is clean and dry, place the two sets of electrodes onto the specific pain site (e.g., neck pain, back pain) and attach the lead wires into the connectors and the TENS unit. With a reliable power source such as batteries or outlet connection, initiate the TENS unit and input the desired settings. Because each device has different settings options, seek the manual or professional assistance in correctly managing the input. Patients can modify settings in accordance with their physician.

Potential Complications

Potential use-dependent analgesic tolerance may occur. This may be offset by N-methyl-D-aspartate blockade, which may prevent tolerance of spinal opioid receptors [30]. Use of mixed frequencies (modulating between high-frequency and low-frequency bursts in the same session) or alternating frequencies (in different sessions) have been shown as a possible tolerance deterrent due to the simultaneous activation of m-opioids and sigma-opioid receptors [22]. Severe adverse effects are rare with most common side effects reported as skin irritation and dermatitis at the electrode placement side.

Repetitive transcranial magnetic stimulation

Overview

rTMS was first described by Baker et al. in 1985 as a non-invasive, non-convulsive neuromodulatory therapy that utilizes alternating magnetic field to induce electromagnetic conduction in the electrical circuitry of the brain [13,32,33]. The user-generated magnetic field influences the electrically sensitive membrane potential of cortical neurons, which can affect the excitability or inhibition of specific neural circuits and may induce long-term potentiation or long-term depression, respectively. The first studies done on this technique were able to produce changes which outlasted the stimulation period [34] and were quickly followed by actual clinical trials for the treatment of major depression [35]. rTMS for the treatment of chronic pain evolved from the efficacy of “implanted electrical stimulation of the motor cortex to treat refractory neuropathic pain,” as reported by Tsubokawa et al. [36]. For central neuropathic pain (CNP), rTMS has been successfully applied to the M1 of the hemisphere contralateral to the pain site. Most recently, Quesada et al. [37] demonstrated an analgesic effect of rTMS on CNP patients while using a longer stimulation period than any other study before it (roughly two months versus one to two weeks). Instead of utilizing the standard daily treatment approach, this study looked at the effects of “4 consecutive rTMS sessions over the course of 2 months.” With an average reduction in pain of 33%, this study provides further support for treating CNP with rTMS, while providing the unique possibility of alleviating the typical clinical burden associated with daily rTMS treatments. In fibromyalgic patients, high-frequency unilateral rTMS of the motor cortex correlating to the right hand and stimulation were able to induce bilateral increases in the patient’s pain threshold [38]. Furthermore, the use of naloxone, an m-opioid receptor antagonist, during the experiment significantly reduced analgesia, suggesting that rTMS on M1 was able to elicit an endogenous opioid effect. Galhardoni et al. [32], studying neuropathic pain patients, noted pain relief of 28% after four sessions of rTMS.

Candidacy

rTMS has been applied broadly with promising results in not only neuropathic pain but also neuropsychiatric conditions (e.g., depression, schizophrenia) and neurological diseases (e.g., Parkinson’s, epilepsy) [39].

Fibromyalgia is present in 2-12% of the population [40] and “is characterized by chronic widespread pain, attentional deficit, nonrestorative sleep, fatigue, and mood instability” [39]. There are numerous evidence-based treatments for this condition which range from tai chi to anticonvulsants, all of which have yielded modest (at best) results. “rTMS studies in patients with fibromyalgia have shown that unilateral rTMS (to the right M1) was able to induce pain relief” [38]. Other studies have shown that rTMS provides significant positive changes to quality of life but no actual changes to pain intensity when compared to a sham study [41]. Furthermore, “it has been shown that right M1 rTMS for 10 consecutive days had a long-lasting analgesic effect, which was more pronounced on the affective components of pain when compared with the reduction of its sensory-discriminative dimensions” [38]. Finally, regular maintenance of rTMS can perpetuate the effect of the initial “daily induction” sessions. Patients who engaged in regular maintenance “not only decreased pain intensity but also improved fatigue, morning tiredness, general activity, walking, and sleep, which are all symptoms of fibromyalgia” [32].

Complex regional pain syndrome (CRPS) type 1 is considered an extremely difficult condition to treat. Patients with this condition suffer from multiple morbidities, including sensory, cognitive, motor, autonomic, humoral, and inflammatory to name a few [42]. Due to the magnitude and sheer breadth of these symptoms, CRPS is highly refractory to traditional therapeutic approaches used in chronic pain. Multiple novel approaches have been utilized on these patients, ranging from specific rehabilitation protocols to ketamine infusions and ablative treatments [43,44]. Two studies have investigated the effects of rTMS on CRPS type 1. The first study compared the analgesic effects of a single session of rTMS to M1 against sham stimulation in a crossover design [45]. “Patients with type I CRPS experienced pain relief 30 seconds after rTMS, which was increased over the next 15 minutes. Over the next 45 minutes, pain relief was decreased” [45]. The second study looked at daily rTMS sessions where all patients underwent identical pre-treatments with rehabilitation and prescription drugs prior to rTMS being applied as an add-on treatment [46]. “Patients were stimulated for 10 consecutive days, and the effects of treatment were assessed for up to 3 months after the last stimulation. Pain was reduced by 51% in the active group compared with 25% in the sham group, and pain relief was maximal after the last stimulation session” [32]. It is both noteworthy and interesting that the patients in this study reported similar effects as those in the previously discussed fibromyalgia study; that is, the effects of rTMS were stronger in relation to the emotional aspects of pain and quality of life versus the sheer measure of the physical quality of pain.

In conclusion, CRPS type 1 and fibromyalgia do not yet have enough strong data to draw specific conclusions as to the efficacy of rTMS as a therapy. However, these initial trials show promise and further studies are certainly worthwhile.

Anatomy

M1 has been the most common site of application for cortical stimulation in patients with chronic pain, with some evidence suggesting that stimulation of the M1 cortex affects distal areas of the brain that process and integrate pain and painful stimuli [32]. Furthermore, stimulation of M1 may induce cerebral blood perfusion in deeper areas of the brain.

Procedure

The procedure can be done continuously at a low frequency (1 Hz) or pulsatile at high frequency (>5 Hz). It is thought that low-frequency rTMS would have inhibitory neuronal effects, while high-frequency rTMS would have excitatory effects [32]. However, several studies have shown that low-frequency rTMS do not provide significant analgesia as opposed to high-frequency rTMS, which had a significant analgesic effect compared to sham stimulation while also yielding some positive effects on cognition [32].

Potential Complications

Possible safety concerns include excessive heating, magnetic displacement of metallic implants, and electrical device malfunctioning [38,47]. Brain tissue heating is minimal and often less than 0.1°C, and it is often protected by high brain-blood perfusion to dissipate heat. Currently, magnetic field exposure of patients undergoing single sessions of rTMS for psychiatric conditions appears to be safe; however, additional prospective studies are required for a firmer conclusion of safety [38]. Most significant and common side effects for rTMS include seizure induction (1.4% risk in epileptic patients), acute hypomania induction, syncope, headache, hearing loss, cognitive changes, and electrode scalp burns. However, in addition to transient headache or localized pain, much of these side effects are rare [47]. Considerations for patient selection include patients who have favorable illness-stimulation interactions that would benefit from targeted cortico-stimulation, illness-specific side effects and risk factors such as psychiatric conditions, and interactions between concurrent treatment such as proconvulsants that decrease the seizure threshold.

Pulsed electromagnetic field

Overview

PEMF has a rich and long history that spans the globe and arrived in the United States as a therapy sometime around World War II [48]. The practice gained real traction in the 1970s when it was deployed using a very specific biphasic low-frequency signal, also known as pulsed radiofrequency electromagnetic field (PRF), for the treatment of unhealed fractures [49]. Roughly 10 years later, PEMF was approved by the Food and Drug Administration (FDA) for “treatment of pain and edema in superficial soft tissues” [48]. As of today, there are multiple systematic reviews which cast some significant doubts on the results of previous trials. In general, this therapy was used in osteoarthritis [50]. More specifically, it was used in knee osteoarthritis [51,52], with doubtful results. When studying the use of PEMF in low back pain, Andrade et al. [53] found several concerns worth mentioning: (1) when compared to standard therapies (physiotherapy, analgesic therapy), there was low/no effect seen with PEMF; (2) there was simply a small effect size seen in many studies; (3) significant heterogeneity was seen between PEMF protocols making it difficult to compare results; and (4) internal validity issues were noted such as (a) intention to treat analysis, (b) randomization concealment, (c) lack of blinding, and (d) failgure to address conflicts of interest. Regardless, as of today, it is accepted that PEMF is capable of initiating various healing processes ranging from delayed fracture healing to multiple sclerosis and Parkinson’s disease [50].

PEMF is another non-invasive neuromodulatory technique that uses electromagnetic fields to induce electrical induction to target specific tissues and has been explored as an analgesic technique [33]. Previous studies have found that PEMF enhances tissue recovery and repair via induction of Eddy currents, which may have an effect in gene expression such as the endogenous opioid pathways [54]. Other proposed mechanisms could involve nitric oxide to improve blood flow, calmodulin, or eicosanoid enzymes [55]. PEMF uses subthreshold, low-intensity, low-frequency waveform, and has been shown to be promising in inflammatory pain such as joint pain in cases of tendinitis, rheumatoid arthritis, and osteoarthritis [56]. Regardless, it must be noted that PEMF is still seen as an adjuvant therapy by the medical community, and its most common use remains in the treatment of a host of musculoskeletal injuries.

Candidacy

PEMF has been considered for various types of pain, including inflammatory pain, diabetic peripheral neuropathies, chronic pain, low back pain, fibromyalgia, and osteoarthritis [33]. Diabetic neuropathy affects roughly 50% of individuals with diabetes, with pain being the most common symptom. Multiple approaches have been applied to address this common complication with the majority being pharmacologic. Pharmacologic approaches bring with them a whole host of issues ranging from habituation and dependence to simple lack of efficacy. PEMF is currently being explored as an option for treating the pain associated with diabetic neuropathy. A study of Graak et al. [57] found that “… available data provide the evidence that PEMF treatment has the potential to modulate neuropathic pain and nerve impulse. It may be due to decrease in endoneural hypoxia, perineural edema, ischemia of peripheral nerves, and improved microcirculation that leads to positive changes after treatment sessions.” The study ultimately urged the use of PEMF as an adjunct therapy with the hope that further high-quality scientific studies would better clarify the issue.

The effects of PEMF on chronic pain (such as chronic low back pain) is as confusing as that of diabetic neuropathy. A study by Nayback-Beebe et al. [58] echoed the results of those done on diabetic neuropathy. That is, chronic low back pain is a significant concern and cost to the healthcare system, PEMF is poorly understood as a tool to address this complex issue, and that in the study PEMF “in addition to usual care produced a significant improvement in self-reported physical health-related quality of life [58]” in patients but also caused a “significant increase in anxiety symptoms” [58]. Therefore, future studies to investigate the effect on PEMF as a treatment are warranted.

Fibromyalgia has demonstrated some significant short-term pain relief with PEMF but it is also plagued by inconsistencies. For instance, Sutbeyza et al. [59] and Paolucci et al. [60] reported studies which demonstrated that both PEMF and “nonpulsed magnetic field delivered by whole-body mats” had significant beneficial impacts on fibromyalgia patients in relation to disease impact and pain intensity. In addition, a randomized, double-blind study done in 2009 by Multanen et al. [54] and another done by Alfano et al. [61] found no improvement in fibromyalgia patients treated with PEMF. It must be noted that there were differences between these studies in terms of dosage and study duration, and both studies by Sutbeyza et al. and Paolucci et al. should be used with caution due to poor sample sizes. However, the truth remains that the jury is still out regarding the efficacy of PEMF in the treatment of fibromyalgia. In summary, the idea that PEMF might induce therapeutic effects in fibromyalgia is simply hypothetical. Other studies worth mentioning have shown that knee osteoarthritis pain could be reduced by 50% along with improvements in stiffness and physical function [24].

Anatomy

PEMF is done locally at a specific site of chronic pain with pads placed adjacent to the site of stimulation [54]. Clinical anatomy is specific to the pathophysiology of the disease process of interest. However, there exist PEMF mats that generate a larger field to envelope the entire body.

Procedure

Commercially available PEMF utilizes low-frequency (<100 Hz) and low-intensity (<1000 Gauss) therapy.

Potential Complications

Because PEMF utilizes subthreshold and low-intensity waveforms, there is higher tolerance and compliance compared to other neuromodulatory devices. Most common adverse reactions reported included musculoskeletal disorders [55]. Some trials exploring PEMF for osteoarthritis reported a few adverse events such as increase joint pain (knee, hip, spine), vomiting, warming sensation, and paraesthesia [56]; however, none of these warranted any adverse event-related dropouts and no significant difference between PEMF and sham stimulation.

Peripheral nerve stimulation

Overview

Peripheral neuropathies are very frequent [62], and can be treated both pharmacologically or non-pharmacologically [2,63]. PNS induces paresthesia via the placement of an implanted electrode over a peripheral nerve (e.g., occipital, sciatic). Although PNS has been around since the 1960s and was introduced alongside procedures such as spinal cord stimulators, it did not gain traction until the late 1990s when the percutaneous approach was developed. This dramatically changed the landscape of the PNS procedure, making it far more efficacious with a profound reduction in its complication profile. For the purposes of this review, we will focus on a discussion of PNS in the outpatient setting for chronic pain. However, it is worth mentioning that the use of PNS in the inpatient setting for acute post-operative pain may be on the rise. However, this discussion is outside the scope of this article.

Candidacy

PNS first gained traction as a therapy for optic neuralgia and a multitude of different headache conditions [64]. However, since the development of the percutaneous approach in the late 1990s, there has been a rapid expansion of this field with intense product development and FDA trials. As a result, we have seen the expansion of PNS into arenas such as (but not limited to): chronic low back pain, focal mononeuropathies, and post-amputation limb pain. Typical candidates for the use of PNS are those who are refractory to other types of therapies and those who have demonstrated a commitment to be a partner in their therapy.

Potentially unique to PNS is the fact that a target nerve must be identified both by the patient and by the practitioner. Other reasons for chronic pain should be ruled out and imaging should support the diagnosis.

Anatomy

PNS implantation involves electrode lead placement adjacent to implicated nerves. In the inpatient setting, point-of-care ultrasound is readily utilized for identifying nerve targets of interest and assisting with procedural placement.

Equipment and Supplies

Recently, non-permanent PNS technologies have gained particular interest. These systems are designed for temporary use and placement occurs as early as the perioperative setting and are typically removed after 60 days [65]. These systems comprise an electrode lead, an external pulse generator, and a remote-controlled operator.

Procedure

While PNS in the outpatient setting often utilizes fluoroscopy, placement in the inpatient setting can be performed with ultrasound guidance and anatomical landmarks. Following placement of the electrode leads, stimulation should be issued to confirm paresthesia of the target nerve. The electrode leads should be anchored to the skin in PNS trials or can be tunneled for more permanent placement. Then, the leads are connected to external pulse generators, which can be internalized for permanent placement if desired, reasonable, and appropriate.

Potential Complications

The minimally invasive nature of this procedure reduces complications. However, there is always the risk of lead migration, site-specific infection, lead breakage/mechanical failure, and/or loss of device efficacy.

Lead migration remains the most common complication. Fortunately, it is now much easier to fix, and a simple technique allows for repositioning without reopening the generator pocket [66,67]. Lead kinks or breaks typically occur at transition points over joints or traversing long distances or are due to excessive stretching or compression.

Infection is an obvious risk with any surgical procedure and PNS is not exempt. Infection rates tend to be approximately 3% [67], although the number can be difficult to nail down. Poor surgical technique is the most likely cause, and the device will likely need to be removed while antibiotic therapy is initiated. The PNS device can be re-implanted after several months.

Limitations

This narrative review has several limitations. The most important are the limited number of the examined neuromodulation techniques. In fact, there are others, for example, the scrambler therapy [68], which were not reviewed. Moreover, for some of the techniques reported in this study, a more extensive revision would be necessary, especially in the light of the incredible evolving indications to their application; for example, for PNS and spinal cord stimulation. Considering its importance, this should be a topic for further study. Lastly, chronic pain represents a significant burden for suffering patients and for the societies who bear the cost of this condition [69], and despite many studies, the understanding of this condition and its etiology remains murky [70].

## Conclusions

Chronic pain poses a significant problem to not only individuals suffering from it but also to societies who bear the cost of this condition. Despite considerable research into chronic pain, the understanding of this condition and its etiology remains uncertain. While the genetics of pain have been a hot topic in recent years, what has also emerged has shown that there is a distinct neural circuit associated with pain that can potentially be disrupted and thus utilized. Neuromodulation techniques leverage these existing neural circuits to create therapies to intervene upon the pathways which carry pain signals. These therapies continue to offer hope to both the patients suffering from these conditions as well as the doctors treating them.

## References

[1] Camilloni A, Nati G, Maggiolini P (2021). Chronic non-cancer pain in primary care: an Italian cross-sectional study. Signa Vitae.

[2] Llampas A, Rekatsina M, Vadalouca A, Paladini A, Varrassi G, Zis P (2020). Pharmacological management of painful peripheral neuropathies: a systematic review [Online ahead of print]. Pain Ther.

[3] Dworkin RH, O'Connor AB, Backonja M (2007). Pharmacologic management of neuropathic pain: evidence-based recommendations. Pain.

[4] Varrassi G, Alon E, Bagnasco M (2019). Towards an effective and safe treatment of inflammatory pain: a Delphi-guided expert consensus. Adv Ther.

[5] Varrassi G, Pergolizzi JV, Dowling P, Paladini A (2020). Ibuprofen safety at the golden anniversary: are all NSAIDs the same? A narrative review. Adv Ther.

[6] Alvaro D, Caraceni AT, Coluzzi F (2020). What to do and what not to do in the management of opioid-induced constipation: a choosing wisely report. Pain Ther.

[7] Sindrup SH, Jensen TS (1999). Efficacy of pharmacological treatments of neuropathic pain: an update and effect related to mechanism of drug action. Pain.

[8] Rekatsina M, Paladini A, Piroli A, Zis P, Pergolizzi JV, Varrassi G (2020). Pathophysiology and therapeutic perspectives of oxidative stress and neurodegenerative diseases: a narrative review. Adv Ther.

[9] Varrassi G, Yeam CT, Rekatsina M, Pergolizzi JV, Zis P, Paladini A (2020). The expanding role of COX inhibitor/opioid receptor agonist combination in the management of pain. Drugs.

[10] Gress K, Charipova K, An D (2020). Treatment recommendations for chronic knee osteoarthritis. Best Pract Res Clin Anaesthesiol.

[11] Wang J, Chen Z (2019). Neuromodulation for pain management. Adv Exp Med Biol.

[12] Kumar K, Rizvi S (2014). Historical and present state of neuromodulation in chronic pain. Curr Pain Headache Rep.

[13] Aamir A, Girach A, Sarrigiannis PG (2020). Repetitive magnetic stimulation for the management of peripheral neuropathic pain: a systematic review. Adv Ther.

[14] Mendonca ME, Santana MB, Baptista AF, Datta A, Bikson M, Fregni F, Araujo CP (2011). Transcranial DC stimulation in fibromyalgia: optimized cortical target supported by high-resolution computational models. J Pain.

[15] Vaseghi B, Zoghi M, Jaberzadeh S (2015). A meta-analysis of site-specific effects of cathodal transcranial direct current stimulation on sensory perception and pain. PLoS One.

[16] Ngernyam N, Jensen MP, Arayawichanon P (2015). The effects of transcranial direct current stimulation in patients with neuropathic pain from spinal cord injury. Clin Neurophysiol.

[17] Bonin Pinto C, Teixeira Costa B, Duarte D (2018). Transcranial direct current stimulation as a therapeutic tool for chronic pain. J ECT.

[18] Thair H, Holloway AL, Newport R, Smith AD (2017). Transcranial direct current stimulation (tDCS): a beginner's guide for design and implementation. Front Neurosci.

[19] Castillo-Saavedra L, Gebodh N, Bikson M (2016). Clinically effective treatment of fibromyalgia pain with high-definition transcranial direct current stimulation: phase II open-label dose optimization. J Pain.

[20] Lefaucheur JP, Antal A, Ayache SS (2017). Evidence-based guidelines on the therapeutic use of transcranial direct current stimulation (tDCS). Clin Neurophysiol.

[21] Potvin S, Marchand S (2016). Pain facilitation and pain inhibition during conditioned pain modulation in fibromyalgia and in healthy controls. Pain.

[22] Johnson M (2014). Transcutaneous electrical nerve stimulation: review of effectiveness. Nurs Stand.

[23] Johnson MI, Mulvey MR, Bagnall AM (2015). Transcutaneous electrical nerve stimulation (TENS) for phantom pain and stump pain following amputation in adults. Cochrane Database Syst Rev.

[24] Kroeling P, Gross A, Graham N (2013). Electrotherapy for neck pain. Cochrane Database Syst Rev.

[25] Hurlow A, Bennett MI, Robb KA, Johnson MI, Simpson KH, Oxberry SG (2012). Transcutaneous electric nerve stimulation (TENS) for cancer pain in adults. Cochrane Database Syst Rev.

[26] Johnson MI, Paley CA, Howe TE, Sluka KA (2015). Transcutaneous electrical nerve stimulation for acute pain. Cochrane Database Syst Rev.

[27] Khadilkar A, Odebiyi DO, Brosseau L, Wells GA (2008). Transcutaneous electrical nerve stimulation (TENS) versus placebo for chronic low-back pain. Cochrane Database Syst Rev.

[28] Vance CG, Dailey DL, Rakel BA, Sluka KA (2014). Using TENS for pain control: the state of the evidence. Pain Manag.

[29] Sluka KA, Bjordal JM, Marchand S, Rakel BA (2013). What makes transcutaneous electrical nerve stimulation work? Making sense of the mixed results in the clinical literature. Phys Ther.

[30] Johnson M (2007). Transcutaneous electrical nerve stimulation: mechanisms, clinical application and evidence. Rev Pain.

[31] DeSantana JM, Walsh DM, Vance C, Rakel BA, Sluka KA (2008). Effectiveness of transcutaneous electrical nerve stimulation for treatment of hyperalgesia and pain. Curr Rheumatol Rep.

[32] Galhardoni R, Correia GS, Araujo H (2015). Repetitive transcranial magnetic stimulation in chronic pain: a review of the literature. Arch Phys Med Rehabil.

[33] Beaulieu K, Beland P, Pinard M (2016). Effect of pulsed electromagnetic field therapy on experimental pain: a double-blind, randomized study in healthy young adults. Electromagn Biol Med.

[34] Pascual-Leone A, Valls-Solé J, Wassermann EM, Hallett M (1994). Responses to rapid-rate transcranial magnetic stimulation of the human motor cortex. Brain.

[35] George MS, Wassermann EM, Williams WA (1995). Daily repetitive transcranial magnetic stimulation (rTMS) improves mood in depression. Neuroreport.

[36] Tsubokawa T, Katayama Y, Yamamoto T, Hirayama T, Koyama S (1991). Treatment of thalamic pain by chronic motor cortex stimulation. Pacing Clin Electrophysiol.

[37] Quesada C, Pommier B, Fauchon C (2020). New procedure of high-frequency repetitive transcranial magnetic stimulation for central neuropathic pain: a placebo-controlled randomized crossover study. Pain.

[38] Passard A, Attal N, Benadhira R (2007). Effects of unilateral repetitive transcranial magnetic stimulation of the motor cortex on chronic widespread pain in fibromyalgia. Brain.

[39] Lefaucheur JP, Aleman A, Baeken C (2020). Evidence-based guidelines on the therapeutic use of repetitive transcranial magnetic stimulation (rTMS): an update (2014-2018). Clin Neurophysiol.

[40] Coaccioli S, Varrassi G, Sabatini C, Marinangeli F, Giuliani M, Puxeddo A (2008). Fibromyalgia: nosography and therapeutic perspectives. Pain Pract.

[41] Boyer L, Dousset A, Roussel P (2014). rTMS in fibromyalgia. A randomized trial evaluating QoL and its brain metabolic substrate. Neurology.

[42] Wasner G, Schattschneider J, Binder A, Baron R (2003). Complex regional pain syndrome - diagnostic, mechanisms, CNS involvement and therapy. Spinal Cord.

[43] Urits I, Shen AH, Jones MR, Viswanath O, Kaye AD (2018). Complex regional pain syndrome, current concepts and treatment options. Curr Pain Headache Rep.

[44] Bussa M, Guttilla D, Lucia M, Mascaro A, Rinaldi S (2015). Complex regional pain syndrome type I: a comprehensive review. Acta Anaesthesiol Scand.

[45] Pleger B, Janssen F, Schwenkreis P, Völker B, Maier C, Tegenthoff M (2004). Repetitive transcranial magnetic stimulation of the motor cortex attenuates pain perception in complex regional pain syndrome type I. Neurosci Lett.

[46] Picarelli H, Teixeira MJ, de Andrade DC (2010). Repetitive transcranial magnetic stimulation is efficacious as an add-on to pharmacological therapy in complex regional pain syndrome (CRPS) type I. J Pain.

[47] Rossi S, Hallett M, Rossini PM, Pascual-Leone A, Safety of TMS Consensus Group (2009). Safety, ethical considerations, and application guidelines for the use of transcranial magnetic stimulation in clinical practice and research. Clin Neurophysiol.

[48] Markov MS (2007). Pulsed electromagnetic field therapy history, state of the art and future. The Environmentalist.

[49] Bassett CA, Pawluk RJ, Pilla AA (1974). Acceleration of fracture repair by electromagnetic fields. A surgically noninvasive method. Ann N Y Acad Sci.

[50] Wu Z, Ding X, Lei G (2018). Efficacy and safety of the pulsed electromagnetic field in osteoarthritis: a meta-analysis. BMJ Open.

[51] Chen L, Duan X, Xing F (2019). Effects of pulsed electromagnetic field therapy on pain, stiffness and physical function in patients with knee osteoarthritis: a systematic review and meta-analysis of randomized controlled trials. J Rehabil Med.

[52] Ay S, Evcik D (2009). The effects of pulsed electromagnetic fields in the treatment of knee osteoarthritis: a randomized, placebo-controlled trial. Rheumatol Int.

[53] Andrade R, Duarte H, Pereira R, Lopes I, Pereira H, Rocha R, Espregueira-Mendes J (2016). Pulsed electromagnetic field therapy effectiveness in low back pain: a systematic review of randomized controlled trials. Porto Biomed J.

[54] Multanen J, Hakkinen A, Heikkinen P, Kautiainen H, Mustalampi S, Ylinen J (2018). Pulsed electromagnetic field therapy in the treatment of pain and other symptoms in fibromyalgia: a randomized controlled study. Bioelectromagnetics.

[55] Sorrell RG, Muhlenfeld J, Moffett J, Stevens G, Kesten S (2018). Evaluation of pulsed electromagnetic field therapy for the treatment of chronic postoperative pain following lumbar surgery: a pilot, double-blind, randomized, sham-controlled clinical trial. J Pain Res.

[56] Iannitti T, Fistetto G, Esposito A, Rottigni V, Palmieri B (2013). Pulsed electromagnetic field therapy for management of osteoarthritis-related pain, stiffness and physical function: clinical experience in the elderly. Clin Interv Aging.

[57] Graak V, Chaudhary S, Bal BS, Sandhu JS (2009). Evaluation of the efficacy of pulsed electromagnetic field in the management of patients with diabetic polyneuropathy. Int J Diabetes Dev Ctries.

[58] Nayback-Beebe AM, Yoder LH, Goff BJ, Arzola S, Weidlich C (2017). The effect of pulsed electromagnetic frequency therapy on health-related quality of life in military service members with chronic low back pain. Nurs Outlook.

[59] Sutbeyaz ST, Sezer N, Koseoglu F, Kibar S (2009). Low-frequency pulsed electromagnetic field therapy in fibromyalgia: a randomized, double-blind, sham-controlled clinical study. Clin J Pain.

[60] Paolucci T, Piccinini G, Iosa M (2016). Efficacy of extremely low-frequency magnetic field in fibromyalgia pain: a pilot study. J Rehabil Res Dev.

[61] Alfano AP, Taylor AG, Foresman PA, Dunkl PR, McConnell GG, Conaway MR, Gillies GT (2001). Static magnetic fields for treatment of fibromyalgia: a randomized controlled trial. J Altern Complem Med.

[62] Latina R, De Marinis MG, Giordano F (2019). Epidemiolgy of chronic pain in the Latium Region, Italy: a cross-sectional study on the clinical characteristics of patients attending pain clinics. Pain Manag Nurs.

[63] Llampas A, Rekatsina M, Vadalouca A, Paladini A, Varrassi G, Zis P (2020). Non-pharmacological management of painful peripheral neuropathies: a systematic review. Adv Ther.

[64] Slavin KV (2011). History of peripheral nerve stimulation. Prog Neurol Surg.

[65] Therapeutics S (2020). SPRINT PNS system: breakthrough treatment for pain.

[66] Slavin KV, Vannemreddy PS (2011). Repositioning of supraorbital nerve stimulation electrode using retrograde needle insertion: a technical note. Neuromodulation.

[67] Monti E (2004). Peripheral nerve stimulation: a percutaneous minimally invasive approach. Neuromodulation.

[68] Sabato AF, Marineo G, Gatti A (2005). Scrambler therapy. Minerva Anesthesiol.

[69] Langley P, Müller-Schwefe G, Nicolaou A, Liedgens H, Pergolizzi J, Varrassi G (2010). The societal impact of pain in the European Union: health-related quality of life and healthcare resource utilization. J Med Econ.

[70] Varrassi G (2020). Pain and postgraduate medicine. Postgrad Med.

